# Supplementary material for: Drosophila melanogaster Mitochondrial Carriers: Similarities and Differences with the Human Carriers
Source: Int J Mol Sci. 2020 Aug 22;21(17):6052. doi: 10.3390/ijms21176052 (PMC7504413; doi:10.3390/ijms21176052)
Supplement: Supplementary file 1 [file ijms-21-06052-s001.pdf]

International Journal of
Molecular Sciences

MDPI

Table S1. List of 48 Drosophila melanogaster putative mitochondrial carriers.

CG1628	CG4994 – Mpcp2	CG7943	CG14209 – Tyler/Shwan
CG1683 – Ant2	CG4995	CG8026	CG16736
CG1907	CG5254	CG8323	CG16944 – sesB
CG2139 – aralar1	CG5646	CG8790 – Dic1	CG18317 – Rim2
CG2616	CG5755 –Slc25A46b	CG8931 – Slc25A46a	CG18324
CG2857 – Tpc2	CG5805	CG9064 – UCP4C	CG18327
CG3057 – colt	CG6492 – UCP4A	CG9090 – Mpc1	CG18340 – Ucp4B
CG3476 – MME1	CG6608 – Tpc1	CG9582	CG18347 – GC1
CG4241 – DPCoAC	CG6782 – sea	CG10920	CG18363 – Dic4
CG4323 – Dic2	CG6851 – Mtch	CG11196 – Dic3	CG18418
CG4743	CG7314 – Bmcp	CG12201 – GC2	CG32103 – SCaMC
CG4963 – mfrn	CG7514	CG14208 – Tyler/Shwan	CG32250 – PMP34
